# Localized palmar angiokeratoma-like lesions following trauma

**DOI:** 10.1016/j.jdcr.2025.03.001

**Published:** 2025-03-14

**Authors:** Alexander J. Jafari, William Sardinia, Paul Bogner, Drew Kuraitis

**Affiliations:** a Department of Dermatology, UTHealth McGovern Medical School, Houston, Texas; b Jacobs School of Medicine, Buffalo, New York; c Department of Dermatology, Roswell Park Comprehensive Cancer Center, Buffalo, New York; d Department of Pathology, Roswell Park Comprehensive Cancer Center, Buffalo, New York; e Department of Dermatology, Tulane University School of Medicine, New Orleans, Louisiana

**Keywords:** acral skin, angiogenesis, angiokeratoma, ibrutinib, malignancy, Waldenstrom’s macroglobulinemia

## Introduction

Angiokeratomas are benign vascular ectasias that arise from the superficial dermis and present as hyperkeratotic violaceous papules. Although the trigger for lesion development may include genetic, medication, or traumatic etiologies,^1^ angiokeratomas commonly present as isolated lesions secondary to trauma. In this report, we describe a patient with Waldenström’s macroglobulinemia treated with ibrutinib who, after a traumatic event, presented with numerous angiokeratoma-like lesions on the palms.

## Case report

**Fig 1:**
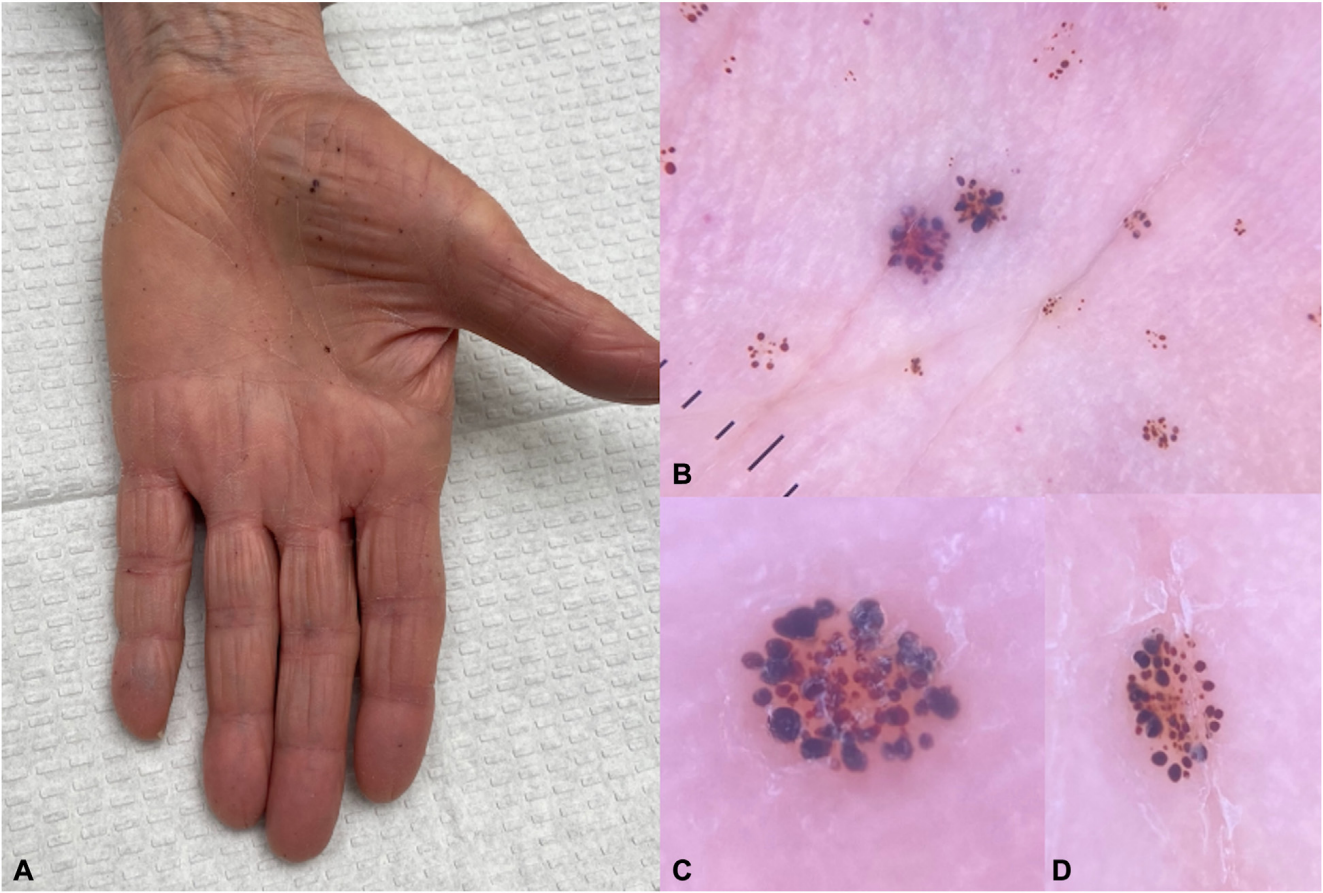
Lesions on the palm at initial presentation (**A**). Dermoscopy of grouped violaceous-to-brawny macules and thin papules (**B**), with apparent formation of lacunae in larger, scaly lesions (**C** and **D**). Notably whitish veils and *white* lined structures are absent.

**Fig 2:**
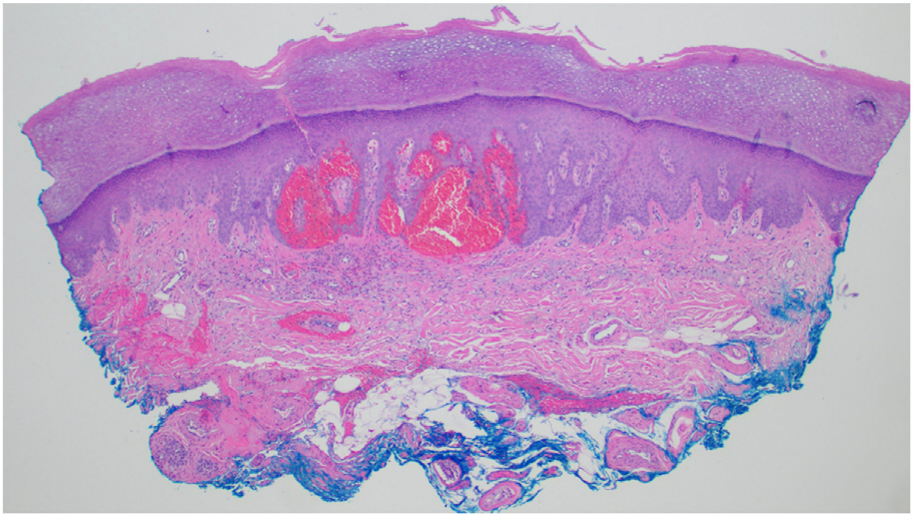
Low-power view of angiokeratoma-like lesion from the palm demonstrating well circumscribed collections of blood originating in the papillary dermis and involving the epidermis (hematoxylin and eosin).

**Fig 3:**
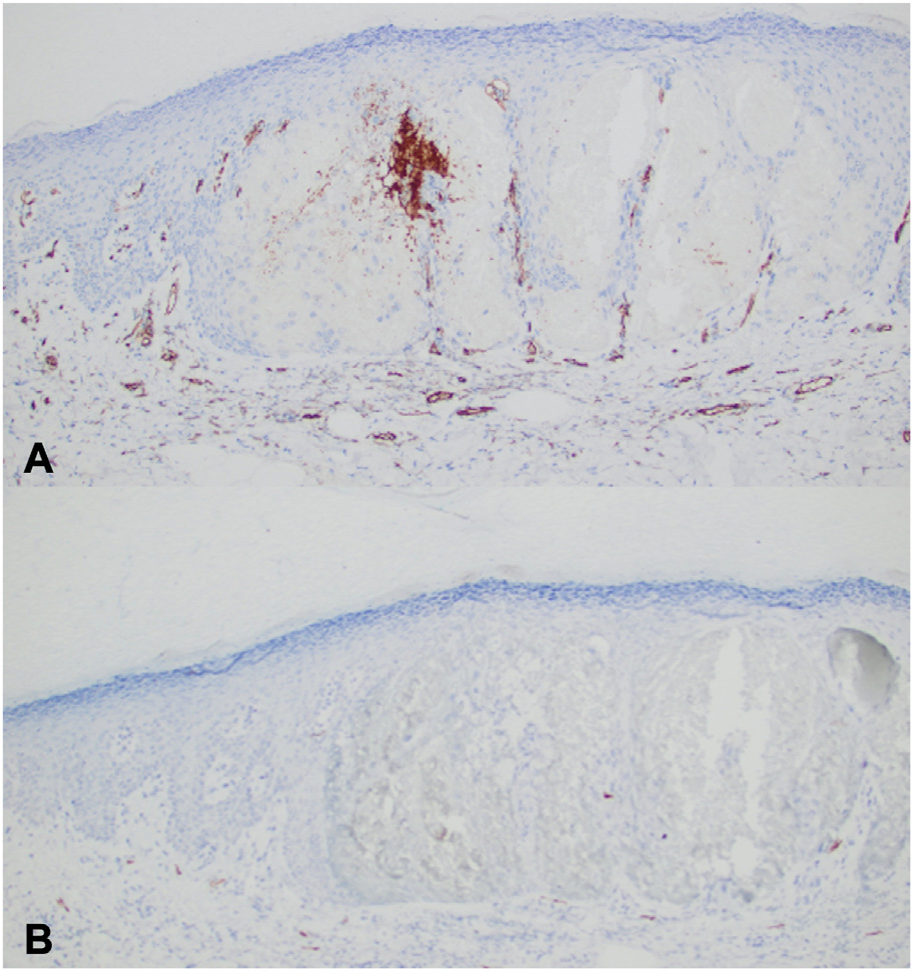
Immunohistochemistry of the angiokeratoma-like lesion presented in Fig 2, demonstrating CD31 (**A**) staining of vessels in the dermis, including at the base of angiokeratoma-like lesions, however failing to stain around the collections of blood in the epidermis. D2-40 (**B**) staining did not demonstrate significant staining in the papillary dermis or epidermis.

A 78-year-old woman presented for evaluation of palmar lesions that appeared shortly after grasping a coniferous tree from a needle-bearing branch 5 months prior. Needles contacting the skin were felt, but no visible injuries were observed at that time. Lesions were asymptomatic, but some were raised and felt rough. The lesions were fixed and did not self-resolve. She had no prior history of similar lesions. Physical examination revealed multiple violaceous-to-brawny macules and thin scaly papules on the palms (Fig 1, *A*), with variable sizes on dermoscopy (Fig 1, *B*) and with purple and red oval structures (Fig 1, *C* and *D*). Her medical history was notable for well controlled Waldenström’s macroglobulinemia, for which she had been taking ibrutinib for over 2 years. Serum vascular endothelial growth factor was within normal limits, at 61 pg/mL. A punch biopsy revealed a vascular lesion at low power with dilated epidermal spaces filled with blood (Fig 2). Immunohistochemistry revealed CD31 staining of the vascular component within the papillary dermis (Fig 3, *A*), but an absence of CD31 staining to the periphery of the epidermal component of the lesion where erythrocytes had collected. D2-40 staining (Fig 3, *B*) was unremarkable. A diagnosis of poorly formed angiokeratoma-like lesions was made.

## Discussion

Palmar angiokeratomas or angiokeratoma-like lesions are rarely reported. In one instance, a woman with mixed lineage acute leukemia undergoing chemotherapy developed painful, palmar angiokeratoma-like nodules approximately 3 weeks into treatment, hypothesized to be chemotherapy-induced or secondary to microtrauma after palmar desquamation.^2^ Although this patient’s lesions were generally consistent with angiokeratoma, the lesions similarly did not stain well with CD31 in the epidermis to define vascular channels, and were termed “angiokeratoma-like,” as true angiokeratomas should have endothelial-lined vascular spaces. This patient’s lesions self-resolved. Another report of angiokeratomas on the palms and soles occurred in a woman with monoclonal gammopathy of undetermined significance.^3^ Histology was consistent with angiokeratoma, but immunohistochemistry was not reported. Our case adds an additional hematologic condition observed in association palmar angiokeratoma-like lesions. Given diversity and scarcity of reports, it is difficult to attribute palmar angiokeratoma-like lesions or cutaneous vascular proliferations to malignancy, but it is interesting that 3 cases in literature are in patients with monoclonal gammopathy of undetermined significance or an underlying hematologic malignancy. These cases are summarized in Table I.

**Table I:** Clinical and histologic characteristics associated with sudden-onset angiokeratomas or angiokeratoma-like lesions in 3 patients with known underlying hematologic conditions

Patient	Age, y	Sex	Clinical presentation	Underlying hematologic condition	Treatment for hematologic condition	Time to onset of cutaneous manifestation(s)	Histopathologic description and immunohistochemistry	Ref
1 (present case)	78	F	Palmar violaceous macules and thin papules	Waldenström’s macroglobulinemia	Ibrutinib (2 year of treatment in total)	Shortly after multiple microtraumas to the palms	• Dilated epidermal spaces filled with blood • CD31 staining of vessels in the dermis but absent around collections of blood in the epidermis • D2-40 (−)	N/A
2	47	F	Palmar angiokeratoma-like purpuric nodules	Mixed lineage acute leukemia	Chemotherapeutic regimen with hydroxyurea, daunorubicin, cytarabine, and methotrexate	3 weeks following initiation of chemotherapy	• Epidermal acanthosis with elongated rete ridges; intraepidermal vascular collections • Endothelial cells not identified nor confirmed with CD31 staining	2
3	40	F	Filiform hyperkeratotic lesions in acral distribution (potential variant of angiokeratoma of Mibelli)	Monoclonal gammopathy of undetermined significance	Not reported	2 year history of these lesions	• Histology consistent with angiokeratoma • Immunohistochemistry not reported	3

In a series evaluating histopathology of 21 solitary angiokeratoma on palms and soles, lesions stained positive for CD31 and CD34, but negative for D2-40.^4^ Our patient’s epidermal lacunae did not stain with CD31, similarly to the patient with leukemia who also presented with acral angiokeratoma-like lesions^2^; however, there was CD31 staining of the vascular component arising from the papillary dermis, reinforcing that the lesions observed were vascular in origin despite not staining throughout the epidermal component as a true angiokeratoma would stain. On dermoscopy, angiokeratomas will commonly display a whitish veil,^5^ and this was also not observed in our patient’s lesions.

Reports of medication-related eruptive angiokeratomas are rare and have mostly been attributed to anticoagulants enoxaparin and heparin.^2^ Our patient was not on an anticoagulant and had been on ibrutinib for more than 2 years. Ibrutinib is a Bruton tyrosine kinase inhibitor that has been reported to induce endothelial cell dysfunction and dysregulate, or inhibit, angiogenesis.^6^ While ibrutinib has not been reported to induce similar lesions, ibrutinib itself is associated with an increased risk of bleeding, as common cutaneous toxicities related to Bruton tyrosine kinase inhibitors may commonly include ecchymosis and petechiae development.^7^ It is likely that the microtraumas initiated vascular lesion formation, and we may consider that ibrutinib’s effect on bleeding allowed for persistence of lesions.

In conclusion, we present a case of palmar angiokeratoma-like lesions in a patient with Waldenström’s macroglobulinemia treated with ibrutinib. While the precise etiology of the patient’s lesions is unclear, trauma was likely the inciting event, and concurrent use of ibrutinib may contribute to lesion persistence. While similar reports are rare, they also include patients with underlying hematologic conditions, possibly representing a common risk factor for this presentation.

## Conflicts of interest

None disclosed.
